# Reprogramming the virulence: Insect defense molecules navigating the epigenetic landscape of *Metarhizium robertsii*

**DOI:** 10.1080/21505594.2017.1421828

**Published:** 2018-03-05

**Authors:** Abid Hussain

**Affiliations:** Laboratory of Bio-Control and Molecular Biology, Department of Arid Land Agriculture, College of Agricultural and Food Sciences, King Faisal University, Hofuf, Al-Ahsa, Saudi Arabia

**Keywords:** Coevolution, Epigenetics, Entomopathogenic fungi, Host-pathogen interaction, Pathogenesis, Virulence

## Abstract

Metarhizium species are the leading bio-control agents well characterized regarding pathogenicity to agricultural, forest, public health, stored grains and urban insect pests. They infect the target host through the tight conidial adherence with the insect cuticle. Conidial binding to the insect cuticle drive the systematic integrated disease development events in target host to impart pathogenesis. However, there is growing evidence that virulence of the pathogen is directly related with proteolytic enzymes including metalloproteinases, chymotrypsin-like proteinases and subtilisin-like proteinases. Successful host pathogenesis is the selection of right set of virulence-related proteinases, which evolved as a result of host-pathogen coevolution.

## Editorial

*Metarhizium* species are the leading bio-control agents well characterized regarding pathogenicity to agricultural [1,2], forest [3,4], public health [5], stored grains [6] and urban [7-9] insect pests. They infect the target host through the tight conidial adherence with the insect cuticle [10]. Conidial binding to the insect cuticle drive the systematic integrated disease development events in target host to impart pathogenesis. However, there is growing evidence that virulence of the pathogen is directly related with proteolytic enzymes including metalloproteinases, chymotrypsin-like proteinases and subtilisin-like proteinases. Successful host pathogenesis is the selection of right set of virulence-related proteinases, which evolved as a result of host-pathogen coevolution [11].

Over the past few decades, host immune responses in the form of anti-fungal molecules against invading fungal pathogens extensively studied to explore host-pathogen interactions [12-16]. There has been relatively little study on the survival of pathogen in the hostile environment of the host. Hence, it is of paramount importance to explore the mechanism driving the pathogen evolution to cope host destructive molecules.

In this issue of Virulence, the authors of the article entitled “The entomopathogenic fungus *Metarhizium robertsii* communicates with the insect host *Galleria mellonella* during infection” designed study to explain reciprocal feedback regulation mechanism by investigating the impact of host defense molecules (antimicrobial peptides and proteinase inhibitors) on the epigenetics of *Metarhizium robertsii* proteolytic enzymes [17]. Prior to spotlight the transcriptional reprogramming regulation in *M. robertsii*, Mukherjee and Vilcinskas determined the immunity-related gene expression in the model host, *G. mellonella*. Here, it is worth mentioning that induction of insect metalloproteinase inhibitors (IMPI) was more modest after infection with avirulent strain (79), reaching only approximately 1.5-fold at 48 h. On the other hand, infection of highly virulent strain (43) confirmed in the supplementary study showed a nearly 9-fold induction of IMPI 4 gene detected 48 h post-infection. This means that virulence of the strain regulate the expression of insect metalloproteinase inhibitors only at the early stage of infection as depicted the findings of Mukherjee and Vilcinskas [17]. However, prolonged infection of virulent (43) and avirulent (79) strain to 9 days on *G. mellonella* larvae exhibited high expression (IMPI 1 gene), reached nearly 113-fold and 97-fold, respectively. Weak induction of IMPIs in response to avirulent strain (79), while time dependent induction of IMPIs by virulent and avirulent strain probably due to the lack of fungal blastospores threshold for the transcription of genes encoding IMPI. Mortality of the larvae in response to the virulent strain might due to the fact that host consumed most of the energy for expression of host defense molecules, and thereby rendering them weak that ultimately lead towards the death of host.

The counterattack of *M. robertsii* to degrade host defense molecules is mediated by expressing selected proteinases that is the outcome of multiple experiments of Mukherjee and Vilcinskas [17]. Firstly, it was made clear the growth inhibition level of each tested microbial peptide and protein inhibitor. Overall, all the tested defense molecules found to affect the germination of both the tested strains. However, trypsin inhibitor followed by metchnikowin tremendously reduced the percent germination of the conidia of virulent (43) and avirulent (79) strain of *M. robertsii*. To assess the involvement of these molecules on the regulation of fungus defense compounds such as extracellular proteinase activity, virulent (43) and avirulent (79) strain of *M. robertsii* were cultured in PDB with host defense molecules. The culturing of virulent strain (43) for 24 h in the presence of metchnikowin led to a significant enhancement of casein based proteinase activity. However, prolonged culturing (72 h) led to a significant reduction in metalloproteinase activity in virulent strain (43) in the presence of metchnikowin as compared to IMPI and lysozyme that showed the highest activity, thus confirming the involvement of these proteinases in pathogenesis by degrading host defense molecules. Further evidence to upregulate pathogenicity related proteins was obtained by quantifying the expression of Chymotrypsin. Results showed that metchnikowin greatly induced the *chy1* expression in both the strains. Overall, six days incubation of virulent strain (43) in the presence of each defense molecule showed comparatively higher expressions of *chy1* to that of avirulent strain (79). The selective upregulations of virulence related genes including *mep1* (metalloproteinase 1), metallopeptidase, and *chy1* (chymotrypsin1) in the virulent strain (43) are the evolved fungal infection strategies that are epigenetically regulated against host defense molecules. Such selective reprogramming not only enhanced virulence [17], but also broad their target host range [18,19].

In their study, Mukherjee and Vilcinskas have further expanded the virulence related epigenetic mechanism of *M. robertsii* by quantifying the genes involved in the regulation of histone deacetylases (HDACs) and histone acetyltransferases (HATs). Results revealed the supremacy of metchnikowin to induce tremendous expression of HDACs and HATs genes in virulent strain (43) of *M. robertsii*. Such inductions are the clear manifestation of epigenetically regulated transcriptional responses to customize the degradation of metchnikowin as evidenced by the LC/MS analysis. It is plausible to suggest that HDACs and HATs like in human pathogens such as *Candida albicans *[**20,21], coordinate the expression of genes involved in the virulence of widely used entomopathogenic fungus, *M. robertsii*.

In summary, we have shown that *M. robertsii* sense the host defense molecules. In order to overcome the hostile environment of the target host, invading fungal pathogens by transcriptomic reprogramming express the genes encoding proteolytic enzymes to degrade the host defense molecules. The refinement of genes required for fungal virulence is the outcome of epigenetically regulated host pathogen reciprocal coevolution mechanism. Future host-pathogen research should focus on the cutting-edge epigenomics technologies that would promise unprecedented insights into the evolutionary arms race between these two adversaries.
